# *In silico* analysis of chimeric TF, Omp31 and BP26 fragments of *Brucella melitensis* for development of a multi subunit vaccine candidate

**Published:** 2014-03

**Authors:** Amir Ghasemi, Reza Ranjbar, Jafar Amani

**Affiliations:** 1 Molecular Biology Research Center, Baqiyatallah University of Medical Sciences, Tehran, Iran; 2 Applied Microbiology Research Center, Baqiyatallah Medical Science University, Tehran, Iran

**Keywords:** Brucellosis, Chimeric protein, Epitope, Vaccination

## Abstract

***Objective(s):*** Brucellosis, especially caused by *Brucella melitensis*, remains one of the most common zoonotic diseases worldwide with more than 500,000 human cases reported annually. The commonly used live attenuated vaccine in ovine brucellosis prophylaxis is *B. melitensis* Rev1. But due to different problems caused by the administration of this vaccine, a protective subunit vaccine against *B. melitensis* is strongly demanded. *Brucella* BP26, Omp31 and TF proteins have shown a considerable potential as protective antigens for brucellosis. Chimeric proteins carrying epitopes or adjuvant sequences increase the possibility of eliciting a broad cellular or humoral immune response. *In silico* tools are highly suited to study, design and evaluate vaccine strategies.

***Materials and Methods: ***In this study, a synthetic chimeric gene, encoding TF, BP26 ^93-111^ and Omp31^48-74 ^was designed. In order to predict the 3D structure of protein, modeling was carried out.

***Results:*** Validation results showed that 91.1% of residues lie in favored or additional allowed region of Ramachandran plot. The epitopes in the chimeric protein are likely to induce both the B-cell and T-cell mediated immune responses.

***Conclusion*** **:** The chimeric protein may be used as multi subunit for development of *Brucella* vaccine candidates.

## Introduction

*Brucella* spp. are Gram-negative and facultative intracellular bacteria which cause brucellosis, a worldwide zoonotic disease leading to abortion in domestic animals and Malta fever in humans (1). *Brucella melitensis *Rev1 is an attenuated smooth strain used to control *B. melitensis *infection, which induces heterologous protection against other* Brucella *spp*. *and is currently considered as the best vaccine for prophylaxis against ovine brucellosis (2). However, due to different problems caused by administration of live attenuated vaccines, a subunit vaccine that is protective against *B. melitensis* is desirable (3). There is an increasing interest in the study of immunogenicity and protective effects of outer membrane proteins (OMPs) of *Brucella* and cytoplasmic proteins (2, 4, 5). TF protein, 485 aa, is a cytoplasmic protein which has also been reported to act as a protective and a good immunogenic antigen (6, 7)*.* BP26 protein, a periplasmic protein, has previously been identified as an immunodominant antigen of the cytosoluble protein extract of *Brucella* in infected cattle, sheep, goats and humans (8). It has been reported that BP26 is a protective antigen (6). Recently, good reactivity of two linear epitope peptides of BP26 (position 93 to 101 and 104 to 111) with 137 *Brucella* infected sheep sera by ELISA has been shown (9). The OMPs of *Brucella *spp. have been characterized as potential immunogenic and protective antigen (Ag) (10, 11). It has been shown that immunization with a peptide that contains amino acids 48 to 74 of Omp31 in adjuvant did not elicit a specific humoral response but elicited a Th1 response mediated by CD4+ T-cells. The peptide in adjuvant also induced levels of protection similar to those induced by rOmp31 against *B. melitensis. *However, it has been shown that recombinant antigens as protective Ags in animal models (3, 6, -) are not called protective response per se, suggesting multi subunit approach should be considered to optimize the immune response. By combining multiple antigens into a single vaccine, broad-spectrum protective immunity against many different clinical isolates has been achieved (21).

In this study, we designed a new structural model containing three putative antigenic determinants of TF, BP26 ^93-111^ and Omp31^48-74^, fused together by hydrophobic linkers. Restriction enzyme sites at 5’ and 3’ ends were added respectively, and codon of this chimeric gene for expression in bacteria was optimized to improve the efficiency of transcription and translation. Finally, a novel *in silico *approach was used to analysis the structure of the designed chimeric protein.

## Materials and Methods

***Sequence analysis***

Sequences of antigens were obtained from GeneBank. In order to identify a fragment common among all sequences, multiple sequence alignments were performed using ClustalW software (http://www.ebi.ac.uk/Tools/clustalw2). A chimeric sequence was constructed by fusing the C terminal of TF, N-terminal of Omp31^48-74^ and middle fragment of BP26^93-111^ using hydrophobic amino acid linkers (22). Stand-alone Leto gene optimization software (www.entelechon.com), OPTIMIZER server (23, 24), Kazusa codon usage database (http://www.kazusa.or.-jp/codon) and Swissprot reverse translation online tool (http://www.bioinformatics.org/sms2/rev_trans.html) were used for the *in silico* gene analysis and optimization of the chimeric gene. The chimeric gene was designed for cloning and expression in *Escherichia coli* (25). VaxiJen server was used to predict the immunogenicity of the whole antigen and its subunit vaccine (26, 27).

***Physicochemical parameters***

Extinction coefficient, theoretical isoelectric point (pI), molecular weight, total number of positive and negative residues, half-life, instability index, aliphatic index and grand average hydropathy (GRAVY), and the physicochemical parameters were computed using Expasy's ProtParam (http://us.expasy.org/tools/ protparam.html) (28).

***Prediction of antigenic B-cell epitopes***

Three web-based B-cell epitope prediction algorithms, Bcepred http://www.imtech.res.in/-raghava/bcepred/, Continuous B-cell epitopes prediction methods based on physico-chemical properties on a non-redundant dataset and Discotope Server http://www.cbs.dtu.dk/services/-DiscoTope/ were used in order to predict discontinuous B-cell epitopes from three dimensional protein structures for analyzing amino acid sequence. Briefly, chimeric proteins were first analyzed for continuous B-cell epitopes using Bcepred and all predicted B-cell epitopes (20 mers) having a BcePreds cutoff score >0.8 were selected. Then the Discotope server was used to predict discontinuous B-cell epitopes. Conformational B-cell epitope from primary sequence was predicted with web server CBTOPE (29). Surface exposed B-cell epitope sequences having cutoff value for BcePreds (>0.8) were selected and analyzed further using VaxiJen (threshold=0.4, ACC output) to check the antigenicity. In addition, location of conformational epitopes on protein surface was defined by Episearch software (30).

***Prediction of T-cell epitopes***

In order to identify common epitopes that bind to both MHC class molecules as well as to count the total number of interacting MHC alleles, Propred-1 (MHC Class-I alleles) (31) and Propred (MHC Class-II alleles) (32) servers utilizing amino acid position coefficients were used. The half maximal (50%) inhibitory concentration (IC50) and antigenicity of common epitopes being predicted by Propred-1 and Propred were calculated using MHCPred server (33) and VaxiJen, respectively.

***Prediction of RNA secondary structure***

Program ‘mfold’ http://www.bioinfo.rpi.edu/-applications/mfold was used to analyze the secondary structure of messenger RNA of the chimeric gene. RNA secondary structure was compared before and after gene optimization. Results were confirmed by other online servers such as CentroidFold Web Server (34).

***Protein secondary structure***

Prediction of secondary structure of the protein was performed by GOR secondary structure prediction method (35). In order for sequence analysis and prediction of protein structure and function such as low-complexity regions, regions lacking regular structure, secondary structure, solvent accessibility, trans-membrane helices, coiled-coil regions, disulfide-bonds, sub-cellular localization as well as functional annotations, PredictProtein server (36) was used .

***Tertiary structure prediction***

Further analysis of the synthetic protein 3D structural stability was done by Swiss-PdbViewer for energy minimization (37). The Swiss model is an automated modeling software developing the 3D structure model of unknown structure protein based on the sequence homology with the known structured protein. In order to visualize the modeled 3D structures, Rasmol tool and Accelrys Discovery Studio 2.5 were used. 

***Tertiary structure validation***

In order to recognize errors in the generated models, their 3D structures were uploaded into

**Figure 1:**
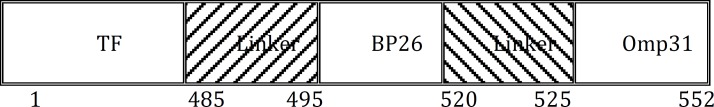
Schematic model displaying the construction of TF, BP26 and Omp 31, bound together by the linkers for expression in prokaryotic cells

ProSA web which is frequently employed in protein structure validation (38). The structure was validated to see the quality of obtained structural stereochemistry by Ramachandran plot in PROCHECK software (39). GROMOS96 implemented in SWISS-MODEL software was used to verify the energy minimization of the model structure (40). The GROMOS96 helps to reduce the bond stretch energy of the modeled protein. It incorporates both bonded and non-bonded forms of energy occupied in the protein molecule.

***Protein solubility prediction***

Solvent accessibility of different residues was evaluated by DSSP and (VADAR) http://redpoll.- pharmacy.ualberta.ca/vadar/.

***Allergenic sites prediction***

AlgPred was used to analyze the presence of possible allergenic sites. AlgPred allows predicating allergens based on the similarity of known epitopes with any region of protein. Further analysis of allergenicity was done by homology search in SDAP database (41).

## Results

***Design and construction of the chimeric gene***

Being reported as strong immunogens (6, 7, 9), TF protein and two peptides of *B. melitensis*, 27 and18 amino acids from Omp31 and BP26, respectively, were selected for the present study. Sequence comparison by ClustalW showed that these sequences were highly conserved among different strains of *B.* *melitensis*. The schematic diagram of protein domain structures with linker's sites is shown in Figure 1. Linkers, consisting EAAAK repeats and expecting to form a monomeric hydrophobic α-helix, were designed and used to separate the different domains. It has been reported that salt bridge Glu--Lys+ between the repeated Ala can stabilize helix formation. Four repeated EAAAK sequences were introduced between different domains for more flexibility and efficient separating. Arrangements of fragment junctions and linker sites are shown in Figure 1.

The amino acid was back translated based on *Escherichia coli* host and the synthetic chimera was analyzed for their codon bias and GC content. Codon adaptation index on the optimized chimeric gene was 0.98. 

Percentage of codon having a frequency distribution of 91–100 in the native chimeric gene was 45% which was significantly improved to 70% in the optimized gene sequence. The overall GC content, which is a measurement of transcriptional and translational efficiency, was improved to 52. Polyadenylation signal, instability elements, and all the *cis*-acting sites that may have a negative influence on the expression rate were removed. Furthermore, the necessary restriction enzyme sites (*EcoRI* and *Hind*III) were introduced at the sequence ends for cloning purpose. 

***Prediction of mRNA structure***

**Figure 2:**
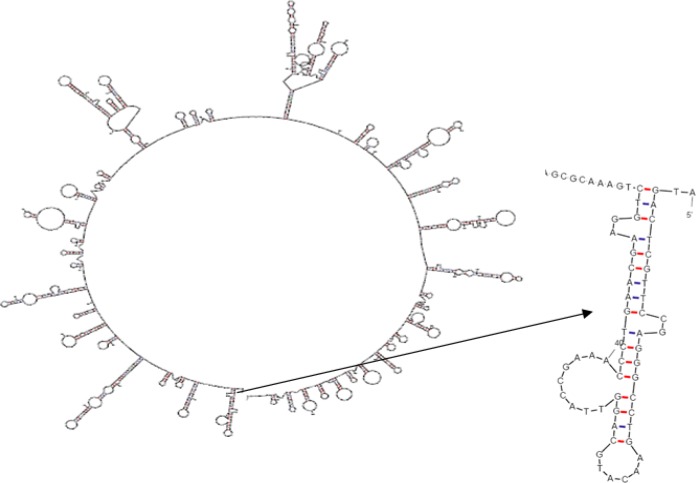
Analysis of mRNA stability and start codon in the structure

In order to determine the potential folding of the chimeric gene, genetic algorithm-based RNA secondary structure prediction was combined with comparative sequence analysis. The 5' terminus of the gene was folded typically as in all bacterial gene structures. Prediction of the minimum free energy for secondary structures formed by RNA molecules was also carried out. All 15 structural elements obtained in this analysis revealed folding of the RNA construct. ΔG of the best predicted structure was −217.92 kcal/mol and the first nucleotides at 5′ did not have a long stable hairpin or pseudoknot (Figure 2) (42).

**Figure 3:**
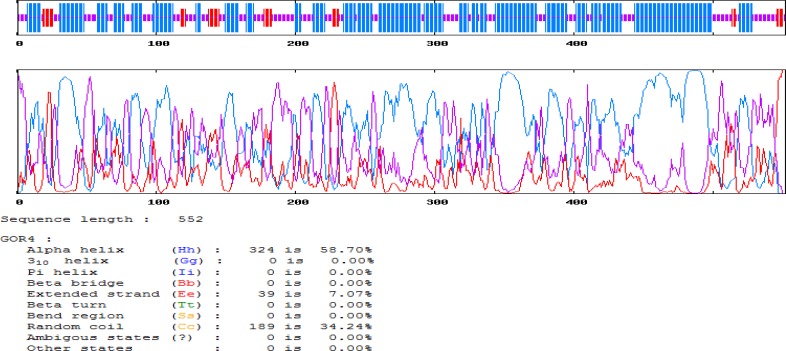
Analysis of chimeric protein secondary structure

***The physico-chemical parameters***

The average molecular weight of synthetic chimeric protein was 61.5 kDa. Isoelectric point (pI) was defined as a pH at which the surface of protein was covered with charge but the net charge of the protein would be zero. The biocomputed half-life was greater than 10 hr. Acidity of the protein was indicated by the pI value, pI: 4.96. On the basis of instability index, Expasy's ProtParam classified the optimized chimeric protein as stable (Instability index: 44.64). Aliphatic index of synthetic chimeric protein was 80.11. Extinction coefficient of optimized chimeric protein at 280 nm was 24870 M^−1^ cm^−1^.

***Secondary structure prediction***

Results showed total residues of 552 which were made up of 39 strands, 324 helices and 189 Random coil. Figure 3 shows secondary structure of fusion protein construction. The results demonstrated that there is a helix located between positions 485-495 and 520-525 that corresponded to the linker fragment (Figure 3). For locating the signal peptide cleavage sites based on neural networks (NN) trained on Gram negative bacteria, SignalP prediction was used. Accordingly, signal peptide cleavage sites were not predicted. 

**Table 1:** Epitope predicted chimeric protein by physico and chemical properties based on Bcepred

Prediction parameters	Epitope segments
Hydrophilic	*ETARGRA, LNDSSRSI, AERNEKSATQPE,MSEDEKEAEKV, SDEEVDEQV,ETKKGKAENEDRVT, EGGADNDAQ,KAGDEKV,LDDETAKK,GRTFEDEETTEEAAREE,YPGQEKE,TDKKVSKEELTAEDEDAASEAKPAK, PKKKAEEGKSEEAEAAA, AGIEDRDLQTG, PDEAAAKN,SSFDKEDNEQVSGS*
Flexibility	*MTRSEG, ERLETAR, EILNDSSRSILAERNEKS, VIMSEDEKEA, KRIASSTRTFETKKGKA, IGLKAGD, GQITRQKVK, FEDEETT, RRYPGQE, NVTDKKVSK, KAAPKKKAEEGKSEE, HPFSSFDKEDNEQVS*
Accessibility	*TRSEGLN, LNEGLKREIKVV, EAKLAERLETARGRARINGFRPGKVPTAHLRKMYGKS, LNDSSRSILAERNEKSATQPE, IMSEDEKEAEKVLDGK, EVKDFSK, SDEEVDEQVKRIASSTRTFETKKGKAENEDRVTI, LKAGDEKV, KVKEVAKPNE, LDDETAKKLGIESLERLRQVVREQIESQYGQITRQKVKRQIL, DGDYQFETPQKLVD, DLQQAGRTFEDEETTEEAAREEYRKLAERRVRLG, VEVTEEELQRAVYDQVRRYPGQEKEIYDFLRRTPDAV, EEKVVDH, INVTDKKVSKEELTAEDEDAASEAKPAKKAAAKKKAAPKKKAEEGKSEEAEA, KEAAAKKKAGIEDRDLQTG, PDEAAAKN, KFKHPFSSFDKEDNEQVS*
Exposed surface	*NEGLKRE, RKMYGKS, LAERNEKSA, MSEDEKEAEKV, VDEQVKRI, RTFETKKGKAENEDR, KVKEVAKPN, DDETAKKL, ITRQKVKRQIL, QFETPQK, AREEYRKLAERR, TEEELQR, YDQVRRYPGQEKEIYD, TDKKVSKEEL, EAKPAKKAAAKKKAAPKKKAEEGKSEE, KEAAAKKKAGIE, FDKEDNEQV*
Polarity	*LNEGLKREIKVV, EAKLAERLETARGRARIN, PTAHLRKMYGKS, RSILAERNEKSAT, IMSEDEKEAEKVLDGK, EVKDFSKIAVTRE, VDISDEEVDEQVKRIA, TRTFETKKGKAENEDRVTID, LKAGDEKV, FDIKVKEVAKPNELVLDDETAKKLGIESLERLRQVVREQIESQ, ITRQKVKRQIL, GRTFEDEETTEEAAREEYRKLAERRVRLGL, VEVTEEELQRAV, RRYPGQEKEIYDF, RAPIFEEKVVDHL, TDKKVSKEELTAEDEDAASEAKPAKKAAAKKKAAPKKKAEEGKSEEAEA, KEAAAKKKAGIEDRDLQT, GGKFKHPFSSFDKEDNEQV*
Antigen propensity	*REIKVVVP, FVFSLNYEVLP, VTREVVD, QLVLGSGQ, KVITVTFP, ERLRQVVREQI, RVRLGLVLSEIG, IFEEKVVDHLL, NIQPIYVYPDE*

**Table 2:** Discontinuous B-cell epitopes prediction based on Discotope server

Residues	Number of residues	Score
*T164, R165, T166, F167, E168, T169, K170, K171, G172, K173, A174, E175, N176, D178, R179, V180, T181, I182, D183, Y184, L185, G186, K187, L188, D189, G190, E191, P192, F193, E194, G195, G196, A197, D198, N199, D200, A201, Q202, F210, I211, P212, G213, F214, Q217, L218, I219, G220, L221, K222, A223, G224, D225, E226, K227, V228, I229, T230, V231, T232, F233, P234, A235, E236, Y237, G238, A239, A240, H241, L242, A243, G244, K245, E246, A247, T248, F249, D250, I251, K252, V253, K254, E255, V256, A257, K258, N260, E261, L262, V263, L264, D265, D266, E267, T268, A269, K270, K271, L272, G273, I274, E275, S276, _:L277, E278, R279, L280*	106	0.762

***Tertiary structure prediction of the fusion protein***

**Figure 4:**
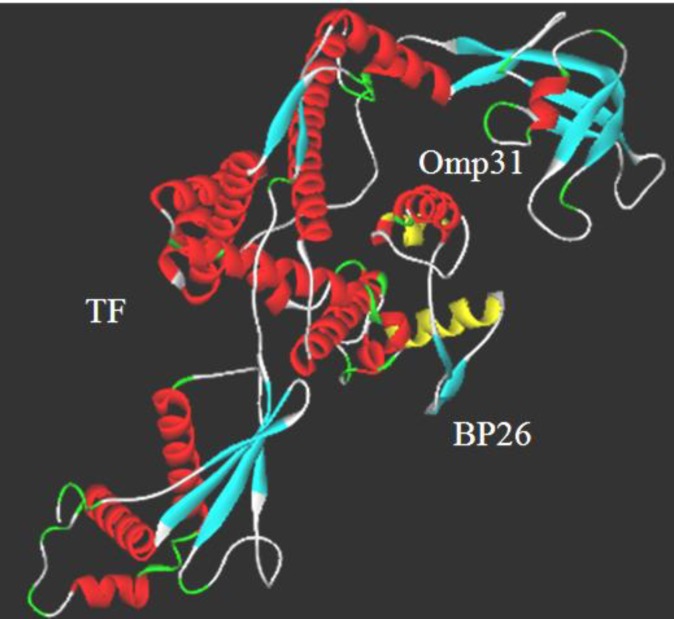
*Ab- initio* and comparative modeling was employed to predict the 3D structure of the chimeric protein, TF-BP26-Omp31. The result was viewed by Accelrys Discovery Studio Visualizer 2.0 software. The linkers are displayed in yellow color

Tertiary structure of the fusion protein was predicted by uploading several models to I-TASSER server (43). Tertiary predication result of the fusion protein construction showed a protein with three domains linked together with linkers. In order to check 3D models of protein structures for potential errors, ProSA was employed. The z-score of the input structure was within the range of scores typically found for native proteins of the similar size (Figure 4). The confidence score (C-score) for estimating the quality of predicted models by I-TASSER was 2.45. C-score is typically in the range of [−5 to 2], where a C-score of higher value signifies the model with a high confidence. In addition, the expected TM-score for this model was 0.54±0.15. The expected RMSD was 10.9±4.6.

***Evaluation of model stability***

**Figure 5:**
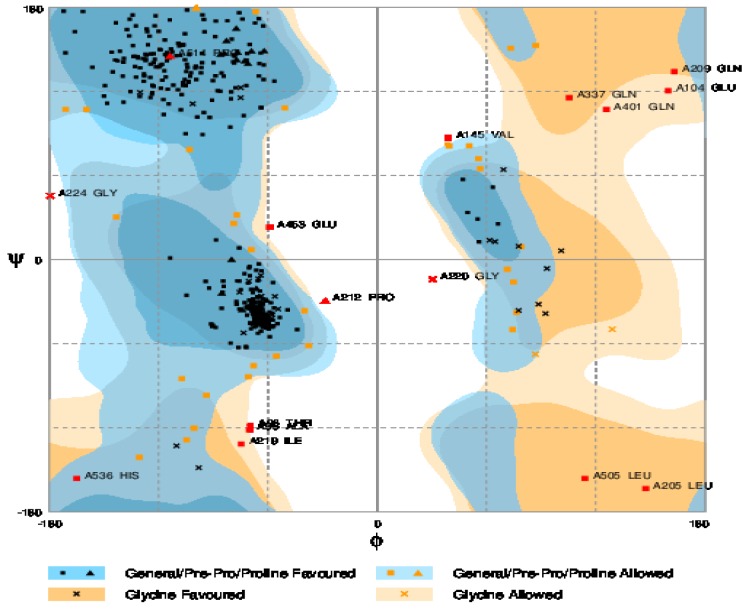
Ramachandran plot for the chimeric protein, TF-BP26-Omp31.Number of residues in favored region (~98.0% expected): 501(91.1%) Number of residues in the allowed region (~2.0% expected): 33 (6.0%) Number of residues in outlier region: 16 (2.9%)

In order to calculate the profile of energy minimization, Spdbv (Swiss-PdbViewer) was used. The amount -14047.257 Kcal/mol indicated that the synthetic chimeric protein had acceptable stability compared to that of original structure of each domain. Additionally, the structural stability of the protein was confirmed by data generation using Ramachandran plot (Figure 5).

***Solvent accessibility prediction***

Major hydrophobic and polarity properties of residual patterns were used to characterize the solvent accessibility distributions. These patterns illustrated that the mean residue accessible surface area (ASA) had given a high solvent accessibility value, approximately fifty percent (Data not shown) (44).

***Antigenic B-cell and T-cell epitopes***

If an antigen is hydrophilic and produces both the B-cell and T-cell mediated immunity, it will be a satisfactory vaccine candidate. Therefore, BCpreds and AAPpreds were used to predict B-cell epitopes of full length protein. The Best epitopes were selected according to the criteria based on cutoff values for BCpreds, AAPpreds and VaxiJen which were >0.8, >0.8 and >0.4, respectively (Table 1). Furthermore, CBTOPE and Discotope servers were used to predict the conformational epitopes of B-cells (Table 2). Each selected B-cell epitope was analyzed for identification of T-cell Propred-I (18 MHC Class-I alleles) and Propred (20 MHC Class-II alleles), by means of MHC Pred to identify the common T-cell epitopes which could interact with both MHC classes I and II with the highest number (Tables 3 and 4).

**Table 3:** The result of prediction for MHCI epitopes

Rank	Sequence	Score	Position
1	*VLDGKADFV*	0.71	112-120
2	*RLRQVVREQ*	0.64	279-287
3	*SLERLRQVV*	0.55	276-284
4	*KLAERRVRL*	0.49	359-367
5	*GINIQPIYV*	0.46	509-517
6	*GLNMQVTET*	0.46	6-14
7	*GLKREIKVV*	0.43	18-26
8	*FLRRTPDAV*	0.43	408-416
9	*VLGSGQFIP*	0.38	204-212
10	*GLKAGDEKV*	0.37	220-228
11	*RLGLVLSEI*	0.36	366-374
12	*ILNDSSRSI*	0.36	78-86
13	*TLNEGLKRE*	0.35	14-22
14	*VTIDYLGKL*	0.33	180-188
15	*GKADFVFSL*	0.26	115-123
16	*KLAERLETA*	0.25	35-43
17	*DLQTGGINI*	0.23	504-512
18	*VLDDETAKK*	0.20	263-271

***Other properties of the construct***

AlgPred tool and SDAP allergen library were used to predict the allergenicity of the sequence. Based on different approaches for allergenicity prediction in AlgPred tool, this protein was not detected as potential allergen. Finally, search for allergens showed no significant similarities between any of the regions and SDAP allergen library.

## Discussion

New strategies are required to protect Brucellosis while avoiding the disadvantages of the currently used live vaccines. Subunit vaccines are an attractive approach for development of effective recombinant vaccines. Although considerable work has been carried out on numerous cell surface and intracellular components, only a few antigens have shown significant protective activity (6, -). Trigger Factor (TF) protein is an ATP-independent chaperone that displays chaperone and peptidyl-prolyl-cis-trans-isomerase activities *in vitro* (48). The positioning of TF at the peptide exit channel, together with its ability to interact with nascent chains as short as 57 residues, renders TF a prime candidate for being the first chaperone that binds to the nascent polypeptide chains (49). TF has also been reported to act as a protective antigen against *B. melitensis* infection (6). It has previously been shown that *Brucella *periplasmic protein, BP26, could be used to distinguish vaccinated livestock from naturally infected animals (50). BP26 is highly conserved among *B. abortus*, *B. suis*, *B. ovis*, and *B. melitensis *(51). Thus, research into this protein has mostly focused on its diagnostic properties in the infected livestock and humans (52). It has been reported that BP26 has vaccine potential when combined with TF. (6). Epitope mapping has been shown that two epitopes of BP26 were very important to its immunogenicity and reaction with infected sheep sera (9). It is also said that the recombinant Omp31 either being associated or notwith *B. ovis *R-LPS, could give an acceptable protection against *B. ovis *infection (53). Immunization with a synthetic peptide comprising residues 48–74 of Omp31 (Omp31^48–74^) has induced a T-helper (Th) 1 response that conferred protection against *B. melitensis*, indicating that this sequence contains a Th1 protective epitope (7). These features attracted our attention to this peptide loop as one potential component for a subcellular vaccine. 

Herein, we designed new construct of *B. melitensis* antigens including TF, Omp31^48–74^ and BP26^93-111^ that contained protective and immunogenic antigens. The usage of linkers is very important in the design of functional chimeric proteins since linkers can play a critical role in displaying specific epitopes in the overall structure of the fusion protein (54). Use of linkers consisting EAAAK repeats and expected to form a monomeric hydrophobic α-helix has been previously described (24). In order to improve the transcription efficiency and transcript stability, codon optimization was performed. This was achieved by improving codon adaptation index and codon frequency distribution, the overall GC content of the gene and removing negative elements that may incorporate unfavorable secondary structures on mRNA. A codon adaptation index of 0.98 was obtained by the optimized gene sequence indicating that the optimized gene sequence can be expressed well. A suitable biased gene would be a codon adaptation index (CAI) of 1.0, although no natural bacterial gene has this theoretical value (24). The average energy minimization was near -14047.257 that were shown in graphical depiction of the predicted minimum free energy for the synthetic gene. Regarding protein structure prediction, the chimeric protein formed three domains that were separated by two main α-helix moieties which can help the protein to form a final structure. These α-helix structures are related to the designation of special amino acid sequences, residues 485-495 and 520-525 inserted between domains. These parts can support the stable structure of a protein which contained three domains. Mfold was one of the softwares used for prediction of RNA secondary structure. Mfold employs a theoretically tractable DP algorithm which can find the minimum ΔG structure within its thermodynamic model and high ability to predict true positive base pairs. Data from mRNA structure prediction showed that the mRNA was stable enough for efficient translation in the host. The physico-chemical parameters of chimeric protein were analyzed. The pI value of protein (pI: 4.96) showed acidic nature of the protein. Extinction coefficient of synthetic chimeric protein at 280 nm was high, indicating the presence of high concentrations of Glu, Lys and Asp. On the basis of instability index, Expasy's ProtParam classifies the synthetic chimeric protein as stable instability index of 44.64. High aliphatic index of the synthetic chimeric protein indicates that the protein may be stable in a wide range of temperature.

**Table 4:** Prediction of MHC II epitopes

Rank	Start position	Residue listing	Score
1	70	*FMAEIVNEI*	326.845
2	359	*KLAERRVRL*	306.550
3	64	*KMYGKSFMA*	233.076
4	312	*YQFETPQKL*	151.142
5	112	*VLDGKADFV*	127.461
6	119	*FVFSLNYEV*	103.580
7	35	*KLAERLETA*	80.345
8	509	*GINIQPIYV*	52.599
9	203	*LVLGSGQFI*	31.581
10	210	*FIPGFEEQL*	25.159
11	101	*IMSEDEKEA*	25.008
12	366	*RLGLVLSEI*	23.995
13	245	*KEATFDIKV*	17.987
14	6	*GLNMQVTET*	17.140
15	370	*VLSEIGEKA*	16.967
16	78	*ILNDSSRSI*	14.543
17	408	*FLRRTPDAV*	11.915
18	122	*SLNYEVLPA*	11.426
19	435	*NINVTDKKV*	9.563
20	227	*KVITVTFPA*	8.824

Both *ab-initio* methods and comparative were used for predicting three-dimensional structure of the chimeric protein. Synthetic chimeric protein has templates in the PDB library on the basis of which the 3D model structure of the protein was generated by Swiss model software.

Our results emphasize that *ab-initio* I-TASSER software can predict the folds as well as good resolution model for synthetic chimeric protein. 

Assessments of reliability and accuracy of experimental and theoretical models of protein structures are necessary. Therefore, the evaluation of the predicted models using both RMSD and TM-score were done. The best RMSD value was the result of our model on template consisting of 552 amino acids; 100% of total protein residues. Expected TM-score of 0.54±0.15 validates the accuracy of the model. A model of correct topology was indicated by a TM-score of > 0.5, its confidence being achieved by other scores including z-score and C-score. The z-score indicates overall model quality and measures the deviation of the total energy of the structure with respect to an energy distribution derived from random conformations. Z-scores, outside of a characteristic range for native proteins, indicat erroneous structures. The ProSA-web results showed that synthetic chimeric protein has features which are the characteristics of native structures. Our chimeric structure showed desirable protein stability based on Ramachandran plot predictions. A negligible 2.9% of the residues were found in Ramachandran plot analysis to be in outliner region that could probably be due to the presence of chimeric junctions. 

An effective vaccine candidate should be able to induce strong B-cell and T-cell responses. For this reason, T-cell and B-cell epitopes mapping are an important method for designing an optimal vaccine. In order to predict linear B-cell epitopes from each protein, BCpreds was employed using a novel method of subsequence kernel. The methods employed for prediction of linear epitopes led to almost identical results with some minor differences. Prediction of conformational epitopes in antibody-antigen interaction is a crucial step for satisfactory design of novel drugs and vaccines (30). Conformational epitopes were predicted with structure-based method and sequence information methods. Our results showed that the structural epitopes derived from CBTOPE and DiscoTope were almost similar.

In this study, the potential T-cell epitopes derived from antigenic B-cell epitopes of chimeric proteins were identified. The selected T-cell epitopes were antigenic with potential to interact with human HLA alleles. Thus, this synthetic chimeric protein had potential to induce both the B-cell and T-cell mediated immune responses. The solubility chance of protein (49.5%) showed that it can be purified under normal conditions when expressed in *E. coli*. Finally, no considerable similarity between any region and SDAP allergen library was observed.

## Conclusion

Our data indicates that epitopes of the synthetic chimeric protein could induce both B-cell and T-cell mediated immune responses which are important for a protective vaccine against Brucellosis. Our data may also suggest this synthetic chimeric protein as a vaccine candidate subunit. Further study is required to establish this notion which is the theme of our future research. 
